# Cystic presentation of primary hepatic neuroendocrine tumour: a case report with a brief review of literature

**DOI:** 10.37349/etat.2023.00133

**Published:** 2023-04-26

**Authors:** Mangesh Londhe, Sakshi Garg, Sushama Gurwale, Charusheela Gore

**Affiliations:** Department of Pathology, Dr. D.Y. Patil Medical College, Hospital and Research Center, Dr. D.Y. Patil Vidyapeeth, Pimpri, Pune 411018, Maharashtra, India; Humanitas Research Hospital, Italy

**Keywords:** Biliary, cystadenoma, primary neoplasm, synaptophysin

## Abstract

Neuroendocrine tumours (NETs) are a rare type of tumours that arise from the neuroendocrine cells which are distributed throughout the body. Of all the gastrointestinal tumours only 1–2% account for NETs. They have an extremely low incidence of 0.17% arising in the intrahepatic bile duct epithelium. Majority of hepatic NETs occur as a result of metastases from the primary NETs. Most cases of primary hepatic NET (PHNET) present as a solid nodular mass. However, predominantly cystic PHNET is extremely rare which mimics other cystic space-occupying lesions clinically and radiologically as seen in this case.

## Introduction

Neuroendocrine tumours (NETs) are a rare type of tumours originating from the neuroendocrine cells which are distributed throughout the body. The gastrointestinal tract and the bronchopulmonary tree are the most preferable sites [1]. Of all the gastrointestinal tumours only 1–2% account for NETs. Primary hepatic NETs (PHNETs) are extremely rare accounting for only 0.3% of all NET cases [2]. The first case of PHNET was reported in 1958 by Edmonson since then less than 200 cases have been reported [1]. They have an extremely low incidence of 0.17% arising in the intrahepatic bile duct epithelium [3]. The majority of hepatic NETs occur as a result of metastases from the primary NETs [4].

Most cases of PHNET present as a solid nodular mass. However, predominantly cystic PHNET is extremely rare wherein, which mimics other cystic spaces occupying lesions clinically and radiologically [1]. We report such a case that was unforeseen clinically and was a histopathological surprise.

PHNETs are slow growing and have the ability to transform into malignancy [3]. They can be clinically diagnosed only in advanced stages [5]. They present with non-specific symptoms, like abdominal discomfort. Only a few cases have carcinoid syndrome [1]. Here, we present a case of cystic PHNET suspected to be biliary cystadenoma preoperatively.

## Case report

A 73-year-old male presented with right upper abdominal pain with bilious vomiting and weight loss for 6 months. The patient had no significant personal, past, or family history. Both general and systemic examination was within normal limits except for hepatomegaly. The complete blood cell count and liver function test were within normal limits. Serology for hepatitis B and C virus were negative.

**Figure 1.**
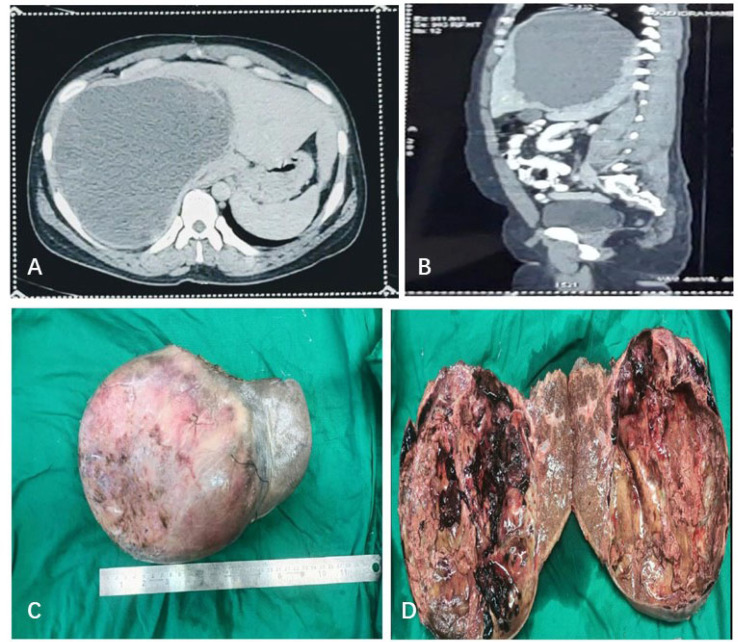
CT and gross. (A) Abdomen (axial view) showing enlarged liver with a well-circumscribed hypoattenuating cystic lesion involving the right lobe; (B) CT abdomen (sagittal view) showing a homogenous nodular enhancement noted along the anterior wall of the cyst; (C) right lobe of the liver with attached cyst with the smooth, intact, and congested external surface; (D) cut surface of the cyst was predominantly cystic, uninoculated, irregular, and haemorrhagic with attached yellowish, friable material. The wall showed focal nodular thickening. The patient underwent partial hepatectomy with cholecystectomy followed by histopathological examination (HPE) of the same

Ultrasonography abdomen pelvis revealed moderate hepatomegaly with a large complex cyst, likely to be a hydatid or haemorrhagic cyst. On computed tomography (CT) of the abdomen and pelvis, the cyst measured approximately 25 cm × 16 cm × 18 cm involving the right lobe and segment IV. A homogenous nodular enhancement was noted along the anterior wall of the cyst measuring 3.9 cm × 1.5 cm in size (Figure 1A and B). The above findings prompted the differential diagnosis of biliary cystadenoma and hydatid cyst. Positron emission tomography and CT showed a large hypodense ametabolic liver mass with a peripheral zone of hypermetabolism. No fluorodeoxyglucose (FDG) avid distant organ involvement uptake was seen. The workup for serum tumour markers like carcinoembryonic antigen (CEA), alpha-fetoprotein (AFP), and carbohydrate antigen 19-9 (CA19-9) was not done as hepatic malignancy was not suspected clinically as well as radiologically.

HPE revealed a part of the right lobe with an attached cyst measuring 18.5 cm in diameter. The surgical margin was 1.5 cm away from the cyst. Externally, the cyst was smooth, congested, and intact. On cutting open thick brownish fluid oozed out, the cyst was solid-cystic and uninoculated. The cyst wall was irregular and haemorrhagic with attached yellowish, friable material. A solid grey area was noted within the cyst which measured 3.0 cm in greatest dimension (Figure 1C and D). The adjacent liver showed tiny whitish nodules ranging from 0.2 cm to 0.4 cm in diameter. The gall bladder was 6 cm in length, externally congested, and showed greenish velvety mucosa.

**Figure 2.**
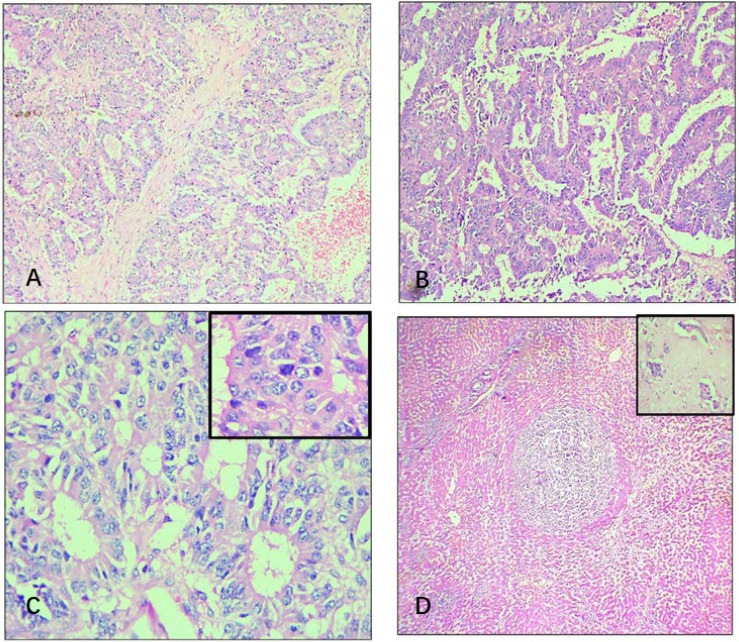
Histopathological findings. (A) Section from cyst wall shows tumour cells arranged in a glandular pattern [hematoxylin and eosin (HE), 100×]; (B) papillary pattern of tumour cells (HE, 100×); (C) salt and pepper chromatin of the tumour cells (HE, 400×, inset-mitoses); (D) satellite nodule of tumour surrounded by normal hepatic sinusoids (HE, 40×, inset-lymphovascular emboli)

The microscopy revealed a cyst with thick fibro collagenous wall beneath which tumour cells were seen arranged in an acinar, glandular, and focal papillary pattern. The individual tumour cells had round to oval nuclei with finely granular chromatin and mild nuclear pleomorphism. The cytoplasm was scant to moderate and eosinophilic. Occasional mitotic activity was noted. Foci of haemorrhage, pigment-laden macrophages, infarction, and cholesterol clefts were noted. Lymphovascular emboli were seen. Sections from the adjacent liver showed multiple satellite nodules tumours with similar histomorphology (Figure 2).

**Figure 3.**
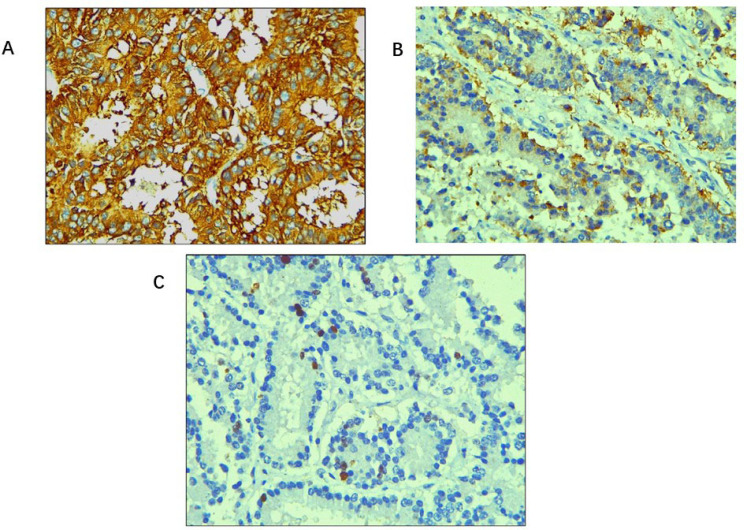
Immunohistochemical stains. (A) Tumour cells showing diffuse and strong cytoplasmic positivity for synaptophysin (IHC, 400×); (B) tumour cells showing positivity for Cg (IHC, 400×); (C) Ki-67 index: 10–12% (IHC, 400×)

The surgical margin was free of tumour. Gall bladder showed features of chronic cholecystitis and was free of tumour. Immunohistochemistry (IHC) workup revealed diffuse and strong positivity for synaptophysin and chromogranin (Cg), and the Ki-67 proliferation index (Ki-67 index) was 10–12% (Figure 3).

All the above findings lead to the confirmatory diagnosis of NET of the liver, grade II with lymphovascular emboli and satellite nodules in the adjacent liver. As a margin-negative resection was achieved, the patient is kept on regular follow-up. To rule out primary *vs.* metastatic NET further workup was advised. Three weeks postoperatively gas chromatograph-1,4,7,10-tetraazacyclododecane-1,4,7,10-tetraacetic acid (GC-DOTA) scan was done which revealed resolution of the previously seen liver mass and no lesion with somatostatin receptors (SSTRs) expression to suggest locoregional residual disease/recurrence or distant organ involvement. Hence, the diagnosis of PHNET was confirmed and metastatic NET was ruled out. The patient is kept on regular follow-up. Serum CgA level done immediately postoperatively was 484 ng/mL (normal < 108 ng/mL) followed by a repeat test done two months later revealing a value of 26.2 ng/mL.

## Discussion

PHNETs are extremely rare accounting for only 0.3% of all NET cases [2]. These are slow-growing tumours that become diagnosable at an advanced stage [5]. They are endocrinologically silent due to the rapid degradation of neoplastic-derived hormones [4]. The aetiology, clinical course and presenting complaints are less known due to their rarity. They show no gender predominance with no known risk factors. Most of the cases present as a solid mass and the presence of a cystic portion makes the diagnosis further challenging. In radiology, they resemble hypervascular hepatic malignancies such as hepatocellular carcinoma (HCC) or intrahepatic cholangiocarcinoma [1]. All the above-mentioned factors make accurate pre-operative diagnosis challenging.

Only 6.8% of PHNETs present as classic carcinoid syndrome with symptoms such as skin flushing, abdominal pain, and diarrhoea [5]. Most cases present as a solid nodular mass. However, predominantly cystic PHNET is extremely rare. In the present case, it was a large cystic mass that led to an erroneous differential diagnosis of a biliary cystadenoma and hydatid cyst. A preoperative biopsy and immunohistochemical markers can help in diagnosis but, the efficacy is controversial [1]. According to Hwang et al. [6], there is a risk of bleeding and tumour seeding with a diagnostic accuracy of 57.1%, thus, a preoperative biopsy not preferred.

Grossly PHNETs vary in size ranging from 1 cm to as large as 20 cm [7]. They are moderately hard mass, yellowish-brown on the cut surface, and well delineated from the surrounding tissue. Cystic lesions are filled with a dark red fluid [2]. On microscopy, they show different patterns such as solid, nested, trabecular, and micro acinar [7]. In our case, glandular and papillary patterns predominated, IHC for markers such as synaptophysin and Cg is effective for identifying a case of PHNETs [4]. Our case exhibited positive staining for both markers. There is a review of cases of PHNETs with predominant cystic change (Table 1).

**Table 1.** Comparison of cases of PHNETs with predominant cystic change

**Various parameters**	**Kim et al. [1]**	**Present case**
Age (years)	51	73
Gender	Female	Male
Clinical presentation	Abdominal discomfort Mild dyspnoea	Abdominal pain Bilious vomiting Significant weight loss
Radiological findings	Left lobe of the liver: cystic; non-smooth; multiseptated mass lesion; occupying hepatic segments II and III; D/D-mucinous cystadenoma	Right lobe of the liver: well-circumscribed hypoattenuating cystic lesion; D/D-biliary cystadenoma; hydatid cyst
Gross	Exophytic tumour 14 cm in greatest dimension Cut surface: solid with varying multiple cysts	Cystic tumour 18.5 cm in greatest dimension Cut surface: predominantly cystic; uniloculated
Microscopy	Tumour cells in a trabecular or cribriform pattern	Tumour cells in an acinar, glandular, and focal papillary pattern
Lymphovascular and satellite nodules	Absent	Present
IHC: Cg; synaptophysin	Both were positive	Both were positive (Ki-67: 10–12%)

D/D: differential diagnosis

PHNETs of the liver are extremely rare and are potential mimickers of other cystic lesions of the liver. Hence, a diagnosis of PHNET should be borne in mind as a rare differential diagnosis for the workup of cystic lesions.

SSTRs expression on neuroendocrine cells has facilitated the diagnosis and treatment of such cases [8]. The octreotide was introduced as a medical therapy for NETs almost 33 years ago [9]. Administration of radiolabelled somatostatin analogue imaging modality such as indium-octreotide is more sensitive and is an upgraded modality for its detection [1]. In this case, a postoperatively GC-DOTA scan was found to be helpful.

Serum CgA level is an excellent method for early detection of recurrence and is highly sensitive and specific. But it cannot differentiate PHNET from other primary NETs. Hence, it is a non-invasive method for checking the recurrence postoperatively [1]. Serum CgA level has been used in this case as a diagnostic tool for follow-up.

Management of these tumours is by surgical resection, with a five-year survival of 74–78%. The prognosis depends on various factors such as size, degree of differentiation, histological grade, Ki-67 index, and metastases [4]. Novel drugs targeting specific pathways within the tumour cells and peptide receptor radionuclide therapy are the options that need to be evaluated in randomized trials [10].

This case highlights a rare presentation of PHNET presenting as a large predominantly, cystic hepatic lesion with the absence of features of carcinoid syndrome, thus making the clinical and radiological diagnosis difficult and challenging.

## Abbreviations

Cg: chromogranin
CT: computed tomography
HE: hematoxylin and eosin
IHC: immunohistochemistry
NETs: neuroendocrine tumours
PHNET: primary hepatic neuroendocrine tumour

## Declarations

### Copyright

© The Author(s) 2023.
